# Brief Virtual Reality and Mixed Reality Mindfulness Breathing Exercise for Emotional Well-Being and Cognitive Functions in University Students: Within-Subjects Experimental Design Study

**DOI:** 10.2196/84239

**Published:** 2026-05-11

**Authors:** Zoey K Y Eun, Charmaine Jiali Koh, Hwajin Yang, Adalia Y H Goh, Meilan Hu, K T A Sandeeshwara Kasturiratna, Andree Hartanto

**Affiliations:** 1 School of Social Sciences, Singapore Management University, 10 Canning Rise, Singapore, 179873, Singapore, +65 68281901

**Keywords:** mindfulness, virtual reality, mixed reality, emotional well-being, working memory capacity

## Abstract

**Background:**

Mindfulness has been shown to enhance emotional well-being and cognitive performance, yet much of this evidence stems from interventions requiring prolonged practice, making them time-consuming and less accessible. Recent studies suggest that brief mindfulness sessions may also yield positive outcomes, but the effectiveness of such interventions in virtual reality (VR) and mixed reality (MR) remains underexplored.

**Objective:**

This study investigates the effects of brief mindfulness breathing exercises delivered through VR and MR on attentional and emotional restoration and self-control capacity.

**Methods:**

Using a within-subjects experimental design, 102 undergraduate participants (n=83, 81.4% female; mean age 20.87, SD 1.89 ) completed a brief (approximately 15 min) VR and MR mindfulness breathing intervention delivered via a head-mounted display and a duration-matched mind-wandering audio control condition. Participants were undergraduates recruited via convenience sampling from psychology courses in a local university in Singapore. These conditions were separated by a 1-week washout period. Emotional well-being and self-control capacity were measured at baseline and post treatment, using self-report measures, whereas working memory capacity was measured at both time points, using operation span at baseline and rotation span post treatment.

**Results:**

Repeated-measures ANOVAs (α=.05) indicated that VR and MR mindfulness breathing conditions significantly enhanced positive affect (*P*<.001, *η_p_*²=0.366), reduced negative affect (*P*<.001, *η_p_*²=0.279), and improved self-control capacity (*P*<.001, *η_p_*²=0.219), compared with the mind-wandering control condition. In contrast, no significant differences were observed for working memory, and Bayesian analyses provided moderate evidence in support of the null hypothesis for both the main effect of condition and the time×condition interaction (BF_01_=7.42 and BF_01_=5.55, respectively). Participants reported significantly greater absorption in the VR and MR conditions than in the control condition (Cohen’s *d*=−1.61, 95% CI −1.91 to −1.32).

**Conclusions:**

These findings suggest that a brief VR and MR mindfulness breathing exercise improves emotional well-being and self-control capacity relative to a mind-wandering control but does not yield short-term benefits for working memory. In contrast to existing studies that typically emphasize stress reduction or rely on multisession digital interventions, this study highlights that a single brief VR or MR session can enhance key emotional and self-regulatory outcomes. As such, these results underscore the potential of VR and MR mindfulness interventions as scalable and accessible tools for promoting mental well-being, while also pointing to the need for further research to optimize their cognitive impact.

## Introduction

### Background

Mindfulness, defined as the enhanced awareness of moment-to-moment experiences, where one intentionally attends to their thoughts, emotions, and bodily sensations in a nonjudgmental and accepting manner [1,2], has consistently been shown to provide wide-ranging psychological benefits across both general [3,4] and clinical populations [5,6]. Evidence from numerous studies indicates that mindfulness-based interventions are associated with a range of positive outcomes related to well-being, including higher levels of life satisfaction [7-9], vitality [7,10], optimism [11,12], pleasant affect [7,13,14], improved emotional processing [15,16], and emotional regulation [17-19]. Furthermore, these practices have been shown to effectively reduce depressive symptoms [7], stress [10,20,21], anxiety [22,23], and emotional reactivity [24-26]. These benefits arise not from suppressing or altering experiences, but from focusing on how individuals interpret their present-moment experiences [27,28]. When individuals perceive their thoughts and feelings as transient mental events that are impermanent in nature, they can reframe negative thoughts and decrease emotional reactivity, fostering a deeper sense of calm and well-being [29-31].

Beyond its mental health benefits, research has increasingly shown that mindfulness enhances cognitive performance [32-35] and is closely associated with improvements in executive functions, including inhibitory control, task-switching, and working memory [36-38]. The strong link between mindfulness and executive functions is theoretically well-grounded, as both rely on maintained attention and cognitive monitoring, indicating an overlap in their underlying cognitive mechanisms [39-41]. By fostering a relaxed mental state that promotes focused attention, mindfulness optimizes resource allocation and reduces mind-wandering, thereby improving cognitive performance [42,43].

In addition to cognitive benefits outlined above, mindfulness is closely linked to self-control. According to self-regulation theory [44], self-control operates as a limited resource that can be depleted under stressful and cognitively demanding conditions and replenished under restorative conditions. Cognitively demanding tasks require long-term executive control, such as maintaining attentional focus on goal-relevant information, suppressing distractions or impulses, and managing competing mental representations, and draw upon limited self-regulatory resources and temporarily reduce subsequent cognitive and emotional control capacity [45]. In contrast, restorative conditions are states or environments that facilitate the recovery of these depleted resources, including exposure to natural environments that promote attentional restoration, engagement in relaxation or mindfulness practices, or periods of rest that foster emotional rebalancing and cognitive recovery. When this resource is depleted, individuals may struggle with goal-directed behavior, impulse control, and regulation. A possible mechanism underlying this effect is that mindfulness enhances two fundamental self-control processes: (1) emotion regulation [17-19] and (2) attention regulation [33,46,47], both of which are crucial for maintaining self-regulatory capacity [44,48]. By enhancing these regulatory capacities, mindfulness provides a potential mechanism through which self-control resources can be replenished, thereby supporting long-term self-regulatory functioning.

Despite accumulating evidence on the positive outcomes of mindfulness on attentional and emotional restoration and self-control capacity, most findings are drawn from intensive programs, which typically span several weeks, requiring participants to engage in regular and often prolonged sessions under expert guidance [49]. Mindfulness-based stress reduction (MBSR) and mindfulness-based cognitive therapy are among the most pervasive and well-established interventions, with participants practicing mindfulness for up to 45 minutes daily and attending weekly group sessions that are 8 weeks long [2,50]. Specifically, 1 study demonstrated that MBSR is associated with reduced stress and enhanced psychological well-being [2], evidenced by improvements in emotional regulation, decreases in anxiety, and increases in life satisfaction among participants. Similar findings have been reported for other intensive mindfulness interventions. For example, a study found that participants who underwent a 1-month intensive mindfulness training reported significant reductions in anxiety and improvements in subjective well-being and self-compassion compared with a waitlist control group [51]. Additionally, another study found that novice meditators who participated in a 10-day intensive mindfulness training retreat demonstrated significant improvements in self-reported mindfulness, depressive symptoms, rumination, working memory, and maintained attention, relative to a comparison group who did not undergo any mindfulness training [32]. Generally, studies have highlighted the efficacy of intensive mindfulness interventions in improving well-being [52,53] and cognitive performance [54,55] compared with control conditions. Mindfulness fosters emotional regulation by increasing present-moment awareness and promoting acceptance of thoughts and feelings. According to the monitor and acceptance theory [56], mindfulness operates through two complementary mechanisms: (1) attention monitoring enhances awareness of moment-to-moment experiences, and (2) acceptance fosters a nonjudgmental and open stance toward these experiences. The interaction between these processes facilitates adaptive emotion regulation by allowing individuals to observe internal states without reacting impulsively.

Despite strong support for intensive mindfulness intervention, research on brief mindfulness has yielded mixed findings. On one hand, some studies report improvements in affect [57], reduced emotional reactivity, and enhanced attentional control [58,59]. Supporting this perspective, 1 study found that a single 15-minute mindfulness session significantly improved positive affect (PA) and reduced emotional reactivity in response to emotionally charged stimuli [60], highlighting the immediate benefits of mindfulness for emotional regulation. Similarly, another study showed that a single 15-minute brief mindfulness session could enhance attentional control by reducing mind-wandering during cognitive tasks [42], suggesting a notable impact on cognitive focus. While mindfulness has been associated with reduced emotional reactivity, it is important to note that reactivity itself is not inherently maladaptive. Emotional reactivity serves adaptive functions, which allow individuals to respond appropriately to meaningful positive or negative events. Excessive dampening of emotional responses, particularly to positive experiences, may reflect emotional blunting or reduced reward sensitivity, which can undermine well-being. Rather than eliminating reactivity, mindfulness promotes adaptive regulation, fostering flexibility to modulate emotions in a context-sensitive manner.

However, the outcomes of brief mindfulness practices are not always positive. Specifically, 1 study observed that a 15-minute mindfulness meditation did not significantly reduce negative affect (NA) relative to active control (guided progressive muscle relaxation training) and passive control (watching a TED Talks video) groups [61]. Likewise, another study found no improvement in working memory capacity following a 15-minute mindfulness breathing exercise in 2 high-powered studies [62]. These mixed findings highlight both the promise and limitations of brief mindfulness and underscore the need for approaches that can deepen engagement without imposing the burden of long-term practice [63,64]. One potential explanation for these inconsistencies is that brief mindfulness exercises may lack the long-term engagement and depth provided by intensive practices, such as MBSR and mindfulness-based cognitive therapy, which are essential for achieving optimal outcomes [49,65]. To address this limitation, recent technological advancements in virtual reality (VR) and mixed reality (MR) may offer promising solutions to enhance brief mindfulness practices.

VR and MR technologies provide avenues for enriching mindfulness practices by offering immersive environments that foster greater focus and engagement [66-68]. For example, one study found that VR provides an immersive environment that may reduce typical barriers to mindfulness practice by increasing engagement and reducing distraction [66]. Another study reported that integrating mindfulness with immersive VR shows promise for improving mood, attention, and engagement [67]. Finally, another study demonstrated that immersive VR conditions can produce greater mindfulness and stress-reduction effects than conventional mindfulness formats in some samples [68]. VR, in particular, provides controlled multisensory environments that simulate natural settings and promote focus [69,70]. Additionally, VR provides multisensory experiences that allow users to interact with virtual natural environments, helping them to disconnect from everyday stressors and deepen their mindfulness practice [71]. Meanwhile, MR, which integrates real and virtual elements, can further enhance mindfulness training by providing real-time feedback and increasing interactivity, which may further boost engagement [72,73]. Recent studies have explored the potential benefits of VR-based mindfulness, showing associations with reduced negative emotions [69,74-76], increased positive emotional states [66,77], and cognitive improvements in attention and working memory [46,78]. Yet, findings have not been entirely consistent, with some studies reporting significant effects on either PA or NA but not both [66,79].

Despite the growing body of research on VR and MR mindfulness interventions, important gaps remain, particularly concerning their effectiveness in brief applications and whether they provide emotional and cognitive benefits comparable with those of more intensive mindfulness practices. Most evidence supporting the cognitive benefits of VR-based mindfulness comes from studies involving multiple sessions, leaving uncertainty about whether shorter interventions yield similar positive outcomes [46,78]. For example, 2 separate studies reported having 8 sessions of the VR mindfulness intervention [46,78]. Moreover, many of these studies face methodological limitations, such as small sample sizes [66] and inadequate or inconsistent control groups [80,81], which constrain the robustness and generalizability of their findings. Research on MR-based mindfulness remains even more limited, despite its potential for enhanced interactivity [73]. These gaps are increasingly salient given recent technological developments, such as the Meta Quest 3 and Apple Vision Pro, which support both VR and MR modes. Emerging mindfulness applications also blend these modalities by embedding virtual cues within real environments or transitioning into full immersion when deeper focus is needed [82]. Thus, addressing these gaps through further empirical investigations is crucial to establishing the efficacy of brief VR and MR mindfulness interventions in enhancing emotional well-being and cognitive functioning and to determining their potential for scalable implementation across diverse real-world settings.

### This Study

This study examined whether a brief mindfulness breathing exercise delivered via VR and MR can enhance emotional restoration, attentional functioning, and self-control capacity relative to a mind-wandering control condition. Using a within-subjects experimental design, participants completed both VR and MR mindfulness breathing exercises as well as a mind-wandering audio condition where participants were instructed to let their mind wander freely with no mindfulness advice provided, with a 1-week washout period between conditions to minimize carryover effects. We hypothesized that a brief mindfulness breathing intervention in VR and MR would lead to significant improvements in attentional and emotional restoration and self-control capacity compared with the mind-wandering control condition. Emotional and self-regulatory outcomes were assessed using standardized self-report measures, while working memory capacity was assessed using 2 well-established complex span tasks—the operation span (OSpan) task and the rotation span (RotSpan) task. These tasks were chosen for their strong validity and sensitivity to within-person changes in attentional functioning [83]. Repeated-measures ANOVA was used to examine condition-based differences across emotional, cognitive, and self-regulatory domains.

In this study, we selected PA and NA as immediate, subjective indicators of emotional well-being to capture participants’ affective states following mindfulness and VR or MR interventions. Although mindfulness has been shown to enhance several executive functions, including inhibitory control, task-switching, and working memory, we focused on working memory as it represents a central component of executive function and reflects the capacity to maintain goal-relevant information during mindful attention [36]. Previous research suggests that mindfulness training can improve working memory capacity by reducing mind-wandering and enhancing attentional stability, making it an appropriate measure for detecting short-term cognitive changes in a brief intervention [42]. We also conceptualized self-control within the limited resource framework of self-regulation, where momentary acts of regulation, attention control, or emotional management draw from a finite pool of control resources [84]. Because self-control sits at the intersection of executive control and emotional regulation [44], it serves as a meaningful link between our cognitive outcome (working memory) and affective outcomes (PA and NA). Hence, this study aimed to examine the effects of a brief (approximately 15 min) VR and MR mindfulness breathing exercise on attentional and emotional restoration and self-control capacity, compared with a mind-wandering control condition.

## Methods

### Transparency and Openness

This study’s design and analysis plans were preregistered. The details of the preregistration are available on AsPredicted [85]. All preregistration documents and supplementary materials are publicly available on ResearchBox #3410 [86]. The study was reported in accordance with the APA Journal Article Reporting Standards (JARS) [87]. Frequentist analyses were conducted using IBM SPSS 29.0.1.0 [88], while Bayesian analyses were conducted using JASP version 0.17.3 [89] to examine evidence for null effects. The extent and pattern of missing data were examined across all primary outcome variables (PA, NA, self-control capacity, and working memory capacity) using SPSS Missing Value Analysis.

### Participants

**Figure 1.**
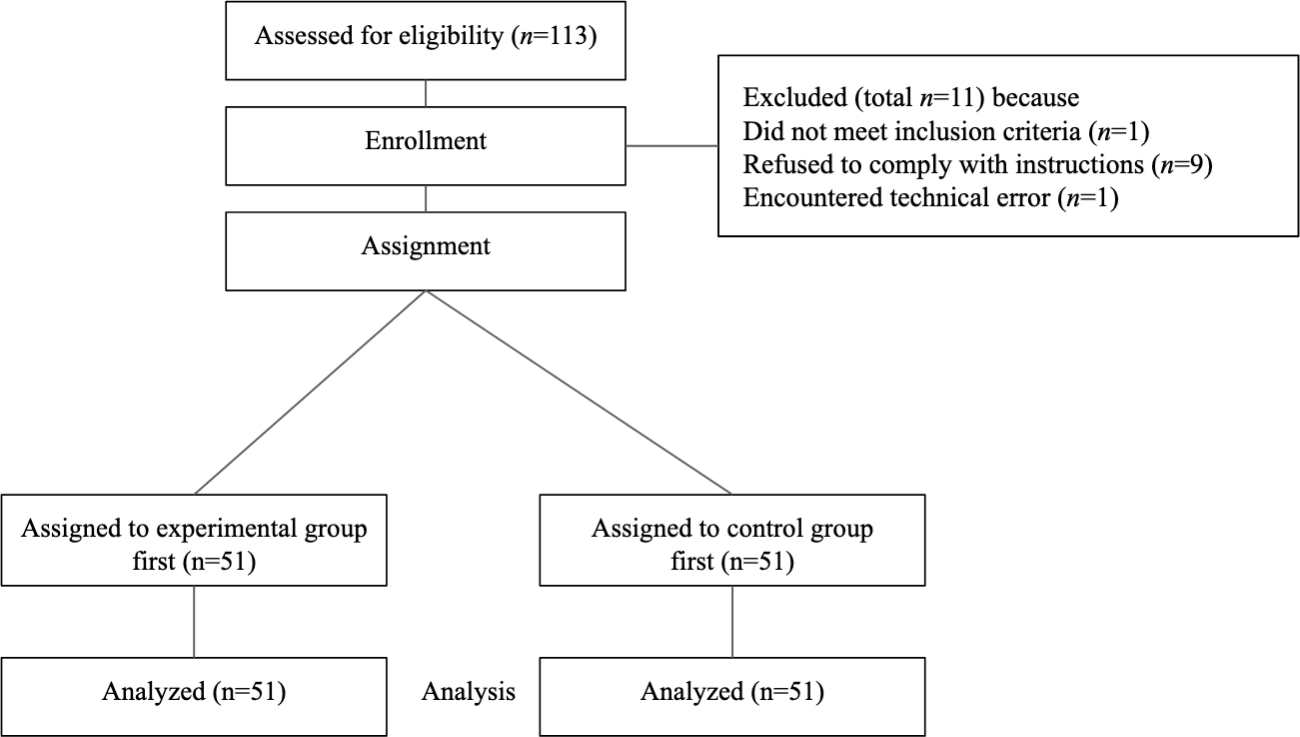
Journal Article Reporting Standards–adapted participant flowchart depicting recruitment, eligibility screening, exclusions, assignment, and final analyzed sample in a within-subjects experiment.

A total of 113 participants were recruited from a local university in Singapore. All participants were psychology undergraduates recruited using convenience sampling, and they received course credits for their participation. Each participant completed 2 counterbalanced sessions. Data from 11 participants were excluded from the analyses due to noncompliance with task instructions or insufficient immersion during either the experimental or control conditions, as indicated by experimenter observation. These participants appeared distracted or failed to engage consistently with the instructions. Specifically, 1 participant, who reported having dyslexia, was removed due to the potential impact of dyslexia on cognitive performance in the RotSpan task. Additionally, 1 participant encountered a technical error, and 9 participants failed to comply with study instructions. This resulted in a final analytic sample of 102 participants. Participant recruitment, eligibility screening, exclusions, assignment, and the final analyzed sample are summarized in a JARS-adapted flowchart in Figure 1. The demographic characteristics of participants are provided in Table 1.

**Table 1.** Demographic characteristics of the sample (N=102) in a within-subjects experiment comparing brief VR^a^ and MR^b^ mindfulness breathing and a mind-wandering control.

Characteristic	Value	Observed range
Sex (female), n (%)	83 (81.4)	—^c^
Ethnicity (Chinese), n (%)	78 (76.5)	—
Age (y), mean (SD)	20.87 (1.89)	18‐30
Monthly household income^d^, mean (SD)	3.70 (1.49)	1‐6
Subjective socioeconomic status^e^, mean (SD)	6.30 (1.42)	2‐9

a VR: virtual reality.

b MR: mixed reality.

c Not applicable.

d Participants rated their monthly household income on a 6-point scale ranging from less than SGD 2000 to more than SGD 20,000; (1) less than SGD 2000 (US $1570.38); (2) SGD 2000-5999 (US $1570.38-US $4710.34); (3) SGD 6000-9999 (US $4711.13-US $7851.10); (4) SGD 10,000-14,999 (US $7851.88- US $11,777.04); (5) SGD 15,000-19,999 (US $11,777.82-US $15,702.98); (6) more than SGD 20,000 (US $15,703.76).

e Participants rated their subjective socioeconomic status using the MacArthur Scale of Subjective Social Status [90], a ladder which represented where people stood in society, and participants had to estimate where one stood on the ladder.

The sample size of this study was determined using an a priori analysis in G*Power 3.1.9.7 [91] to ensure that the study was designed to meet the minimum sample size needed to detect an effect with 80% statistical power. Based on a medium effect size of *f*=0.25, an α level of .05, and at least 80% power (1–β), the analysis indicated that a minimum of 34 participants would be needed. Additionally, to ensure sufficient statistical power, a post hoc sensitivity power analysis was conducted using the same G*Power settings. This analysis indicated that with our final sample size (n=102), the study was adequately powered to detect the time×condition interaction effects specified by the study design as small as *f*=0.14 (equivalent to *η_p_*^2^=0.019), which represents a small-to-medium effect size. No missing values were observed for any of the primary outcome variables.

### Ethical Considerations

**Figure 2.**
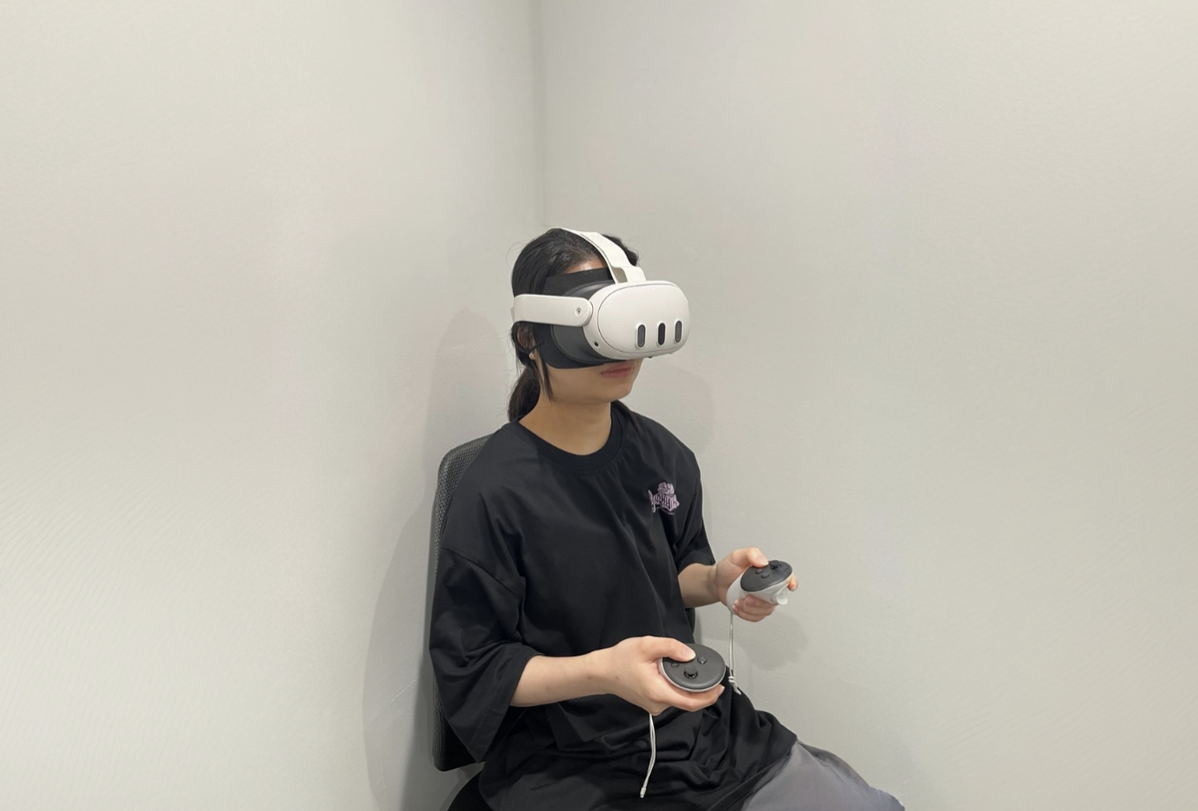
Participant wearing a Meta Quest 3 VR headset and hand controllers during the virtual reality and mixed reality mindfulness breathing condition in a within-subjects experiment.

All procedures were approved by the university’s Institutional Review Board (IRB-24‐119-A087-M2(1124)) and complied with its ethical guidelines. Participants were informed about the study aims, procedures, potential risks, and their right to withdraw at any time without penalty and provided written informed consent before the first session. To protect privacy and retain confidentiality, participants were identified only by unique identification codes. No personally identifiable information was stored with the research data. All analyses were conducted using deidentified datasets, with data access restricted to the research team. Participants received course credit in exchange for completing both study sessions. No images included in the manuscript or supplementary materials allow for the identification of individual participants. Informed consent was obtained from the individual depicted in Figure 2 for the use of their image in this publication.

### Study Design

This study used a 2 (condition: VR+MR mindfulness breathing vs mind-wandering)×2 (time: baseline vs postintervention) within-subjects experimental design. The within-subjects approach was chosen to control for interpersonal variability, thereby reducing errors associated with individual differences and enhancing the ability to detect true differences between conditions, ultimately increasing statistical power. All participants experienced both the experimental and control conditions. In the experimental condition, participants engaged in a brief VR and MR mindfulness breathing intervention that lasted approximately 15 minutes. In the control condition, they engaged in a mind-wandering audio that lasted approximately 15 minutes. The order of conditions was counterbalanced, with half of the participants completing the experimental condition first and the other half completing the control condition first. To mitigate potential carry-over effects, sessions were separated by a 1-week interval, which served as a washout period to allow any practice or residual effects from the first session to subside [92,93]. Participants underwent random assignment through Qualtrics to establish the sequence in which they experienced the 2 different conditions.

### Materials

#### Emotional Well-Being

State affect was measured using the 18-item Circumplex Model of Affect Scale [94], which evaluates emotional states along 2 independent dimensions—PA and NA. Participants rated their current emotional state on a 5-point Likert scale (1=*Not at all*, 5=*Extremely*) in response to the question, “Overall, how do you feel right now?” The PA scale includes 9 items, that is, energetic, enthusiastic, excited, happy, cheerful, pleasant, calm, content, and relaxed. The NA scale includes 9 items, that is, angry, hostile, irritable, nervous, anxious, tense, dejected, sad, and unhappy. Higher scores indicated greater agreement with the respective states. The PA scale demonstrated good internal consistency at baseline (α_pre_=.90), after the VR or MR mindfulness condition (α_post_=.93), and after the mind-wandering control condition (α_post_=.91). The NA scale demonstrated good internal consistency at baseline (α_pre_=.90), after the VR or MR mindfulness condition (α_post_=.88), and after the mind-wandering control condition (α_post_=.86).

#### Self-Control Capacity

Self-control capacity was measured using the 5-item Brief State Self-Control Capacity Scale [95]. Participants rated their agreement with each statement (eg, I feel drained now; I feel calm and rational now) on a 7-point Likert scale (1=*Very untrue of me*, 7=*Very true of me*), in response to the question, “Overall, how do you feel right now?” Higher scores indicated greater agreement with the respective states. The scale demonstrated good internal consistency at baseline (α_pre_=.75), after the VR or MR mindfulness condition (α_post_=.74), and after the mind-wandering control condition (α_post_=.78).

#### Working Memory Tasks

Working memory capacity was measured using 2 established shortened complex span tasks—OSpan task and the RotSpan task, administered via E-Prime 3.0 [96]. These tasks are accessible on the Attention & Working Memory Lab website [97]. These tasks were selected as performance on complex span tasks has been shown to be malleable and sensitive to experimental manipulation such as training instructions [83]. Although the OSpan (verbal) and RotSpan (spatial) tasks differ in stimulus modality, both tasks are validated measures of domain-general working memory capacity and share the same dual-task structure, which makes them comparable indicators of working memory capacity.

In both tasks, participants were required to memorize a series of items (eg, letters or arrows) while performing interspersed distractor tasks (eg, solving math equations or identifying rotated letters). The number of items to be memorized varied per trial, ranging from 2 to 7, with each sequence length appearing 3 times in a randomized order. To minimize the likelihood of rehearsing memorized items during the distractor task, participants were required to respond to the distractor tasks at a steady pace. Before beginning the main trials, participants completed a practice round for each task to ensure they fully understood the instructions.

**Figure 3.**
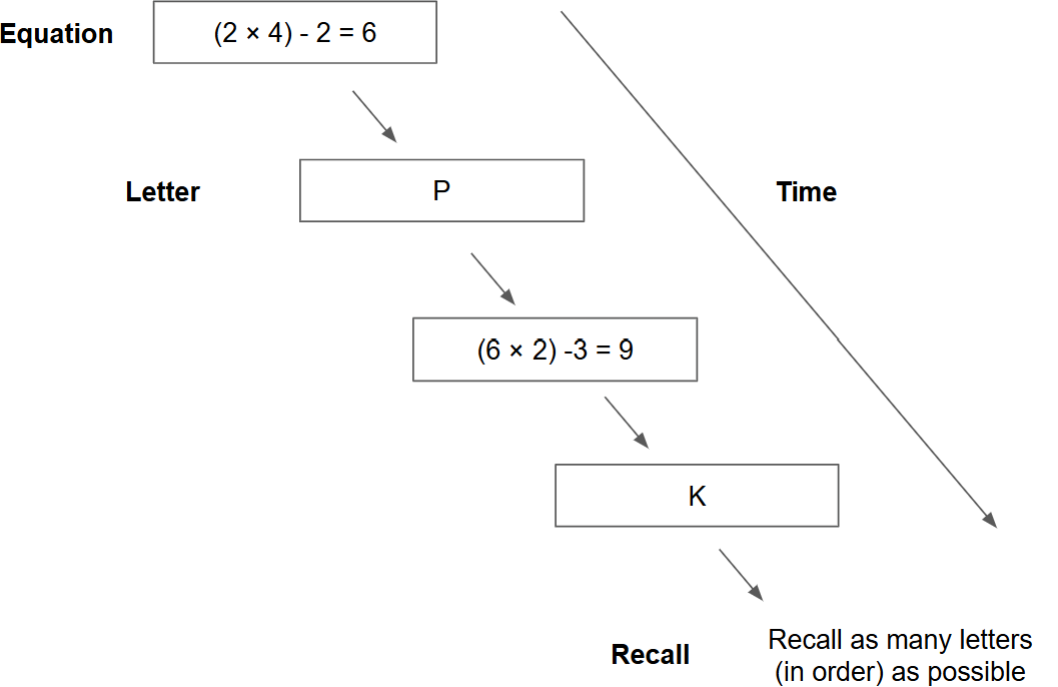
Operation span task flow to assess working memory capacity in an experimental within-subjects trial in undergraduate students in Singapore.

Baseline working memory capacity was assessed using the OSpan task. In this task, participants solved mathematical equations while simultaneously memorizing a sequence of letters for later recall (Figure 3). The letters served as items to be remembered, and the math problems acted as distractors. During each trial, participants first solved a math equation and then viewed a letter. This math-letter sequence was repeated between 3 and 7 times, with the number of repetitions varying unpredictably across trials. At the end of each trial, participants were asked to recall the letters in the correct order. Performance was scored using the partial credit unit method, which is based on the number of correctly recalled letters divided by the total number of letters presented within each trial and then averaged across all trials [83]. Each participant completed 2 blocks of trials.

**Figure 4.**
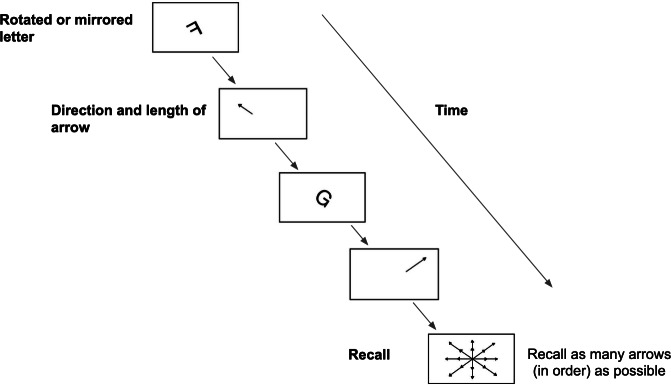
Rotation span task flow to assess working memory capacity in an experimental within-subjects trial in undergraduate students in Singapore.

Posttreatment working memory capacity was measured using the RotSpan task, which is similar to the OSpan task but involves different items and operations. In this task, participants judged whether a rotated letter was correctly oriented or mirrored while also memorizing a sequence of arrows, each varying in direction and length (Figure 4). The arrows served as items to be remembered, and the rotated letters acted as distractors. During each trial, participants first judged the orientation of a rotated letter and then viewed an arrow. The rotation-arrow sequence was repeated 2-5 times, with the number of repetitions varying across trials. After each sequence, participants were required to recall the arrows in the correct order. Each participant completed 2 blocks of trials. The partial credit unit score was used to index performance [83].

### Brief VR and MR Mindfulness Intervention

In the VR and MR mindfulness breathing condition, participants engaged in a mindfulness breathing exercise that lasted approximately 15 minutes using the Meta Quest 3 VR headset (Figure 2). Before the mindfulness breathing exercise, the experimenter instructed participants to prepare and explained how to use the controllers for 2 specific functions, which were used to navigate the virtual environments. Participants were informed that their VR screen would be shared on an external monitor (but not recorded) and reminded to follow the guided audio throughout the exercise. The VR and MR mindfulness breathing condition was administered through the Headspace XR app. Headspace XR offers both virtual and MR experiences, featuring mood-enhancing games, personalized guided meditations, and exercises designed to help users improve their mind-body connection through movement and breathing techniques. For this study, participants were tasked to engage in 3 mindfulness breathing activities within Headspace XR.

**Figure 5.**
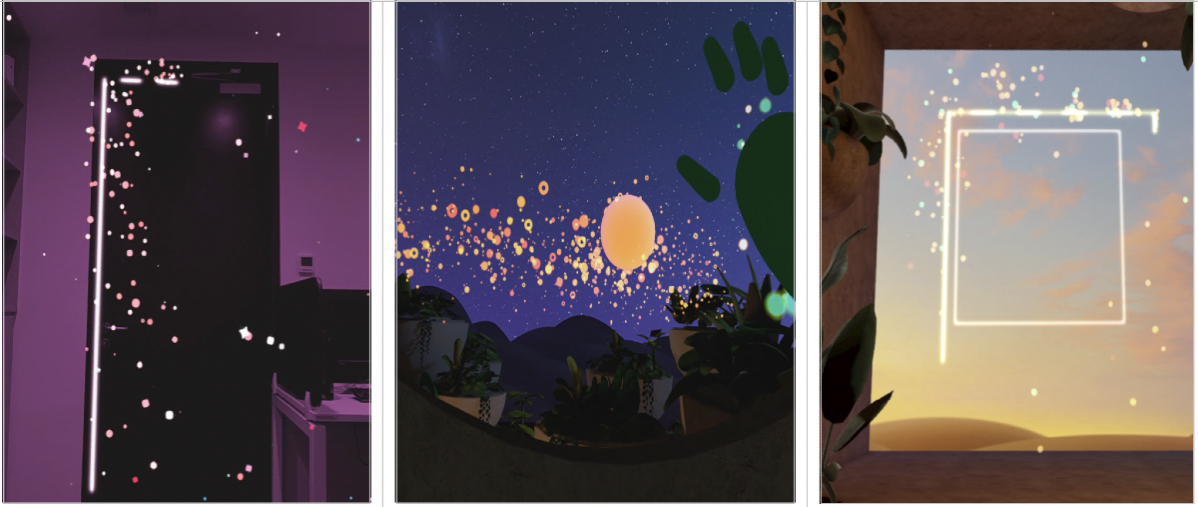
Screenshots of the three mindfulness breathing activities used in the virtual reality and mixed reality conditions. The 3 panels depict the mindfulness breathing environments used in the virtual reality and mixed reality conditions. From left to right: Take a Beat Portal, Firefly Treehouse, and Boxy Treehouse.

The first activity, “Take a Beat Portal,” adopts a technique known as “box breathing,” which takes place in an MR setting (Figure 5). In this activity, participants would first find an object or feature in the room that is shaped in a square. Then, they were instructed by the audio to draw a box-shaped virtual overlay that they would be using for the breathing exercise. Once the shape is drawn, participants complete three sets of four controlled breathing cycles. The guided audio included instructions, such as “Inhaling…feeling your chest and stomach expanding [...] Holding…feeling the breath in your body [...] Exhale… feeling the release of any tension [...] Holding… before the next breath.” This entire activity lasts approximately 5 minutes.

The second activity, “Firefly Treehouse,” is a breathing exercise accompanied by arm movements (Figure 5). This activity takes place in a virtual setting where participants are guided to take deep breaths, raising their arms while inhaling and lowering them during exhaling. The guided audio included directions, such as “Take in a deep breath, and as you inhale raise your hands up above your head [...] Hang there for a moment and as you exhale, bring your hands back to your side.” This entire activity lasts approximately 3 minutes.

The third activity, “Boxy Treehouse,” is a box breathing exercise similar to the first activity, with the only difference being that it is carried out in a VR setting (Figure 5). In this box breathing exercise, participants would similarly undergo 3 sets of 4 breathing exercises. The guided audio also included similar instructions as the first activity, such as “Inhaling…feeling your chest and stomach expanding [...] Holding…feeling the breath in your body [...] Exhale… feeling the release of any tension [...] Holding… before the next breath.” Throughout all 3 activities, participants were instructed to direct their attention to the present moment, feeling their breath, and being aware of each of their breaths. This entire activity lasts approximately 5 minutes.

### Mind-Wandering Control

In the mind-wandering condition, participants listened to a 15-minute audio track that allowed for free mind-wandering. Participants listened to the audio using headphones on the desktop. It included instructions, such as “Now we’re going to do an exercise for 15 minutes [...] Now simply think about whatever comes to mind, let your mind wander freely without thinking about anything in particular [...] Let your mind roam as it normally would [...] Allow your thoughts to wander wherever they may go [...] Go ahead and follow whatever thoughts that come to mind [...] Continue letting your mind wander, allowing your thoughts to wander wherever they may go.” Throughout the mind-wandering audio, there were no mindfulness instructions or interventions given.

### Procedures

The within-subject experiment (VR+MR mindfulness breathing vs mind-wandering) consisted of 2 sessions held 1 week apart in the laboratory. Data collection spanned 13 weeks during the semester, and analysis commenced only after the final session was completed. Participants were randomly assigned to either the VR and MR mindfulness breathing experimental condition or the mind-wandering control condition during their first session and assigned to the other condition in the second session, a week later. Throughout the study, the experimenter monitored participants to maintain data quality.

At the start of each session, participants received a link to the Qualtrics survey and were directed to the informed consent page. Participants used a desktop computer with headphones and a VR headset to complete the study. After providing informed consent, each participant generated a unique personal ID to be used across both sessions. Participants began each session by completing baseline measures, including the Circumplex Model of Affect, SMS-5, and OSpan. Following the baseline assessments, participants proceeded to complete activities in their assigned conditions.

**Figure 6.**
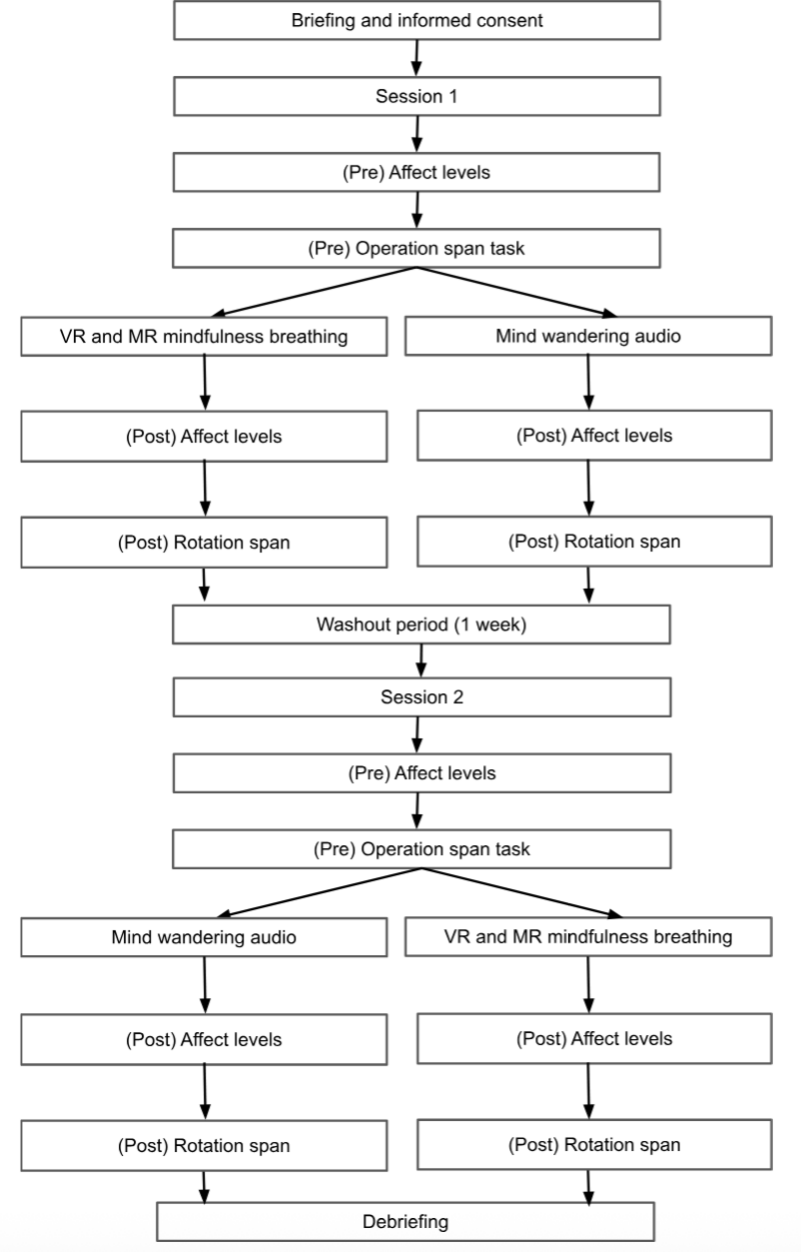
Flowchart of the study procedure illustrating the counterbalanced within-subjects design comparing brief virtual reality and mixed reality mindfulness breathing and a mind-wandering control condition. Total sample size was n=102. MR: mixed reality; VR: virtual reality.

After undergoing their respective conditions, a manipulation check was conducted to assess whether the participants were absorbed during the VR and MR mindfulness breathing intervention [42,98,99]. Participants rated the extent to which they felt absorbed in the present moment, focused on their breathing, and the physical sensations of their breathing. The 3 questions were asked using a 7-point Likert scale (1=*Not at all absorbed*, 7=*Extremely absorbed*), where higher scores correspond to greater levels of absorption. A manipulation check consisting of the same questions was conducted for the mind-wandering condition. Finally, they completed posttreatment measures, including the Circumplex Model of Affect, SMS-5, and RotSpan. Participants filled in their demographics at the end of the first session. At the end of the second session, participants were debriefed about the purpose of the study (Figure 6).

## Results

### Manipulation Check

In line with our preregistration, we conducted a paired *t* test analysis, which revealed a significant difference in absorption between conditions, *t*_101_=−16.29, Cohen *d*=−1.61, 95% CI −1.91 to −1.32; *P*<.001, indicating that participants reported greater absorption in the VR and MR mindfulness breathing condition (mean 15.46, SD 3.32) than in the mind-wandering control condition (mean 9.11, SD 4.07). Bayesian analysis provided extreme evidence in favor of the alternative hypothesis, BF_10_>100 [100], indicating a substantial difference in absorption levels between conditions.

### Working Memory Capacity

**Figure 7.**
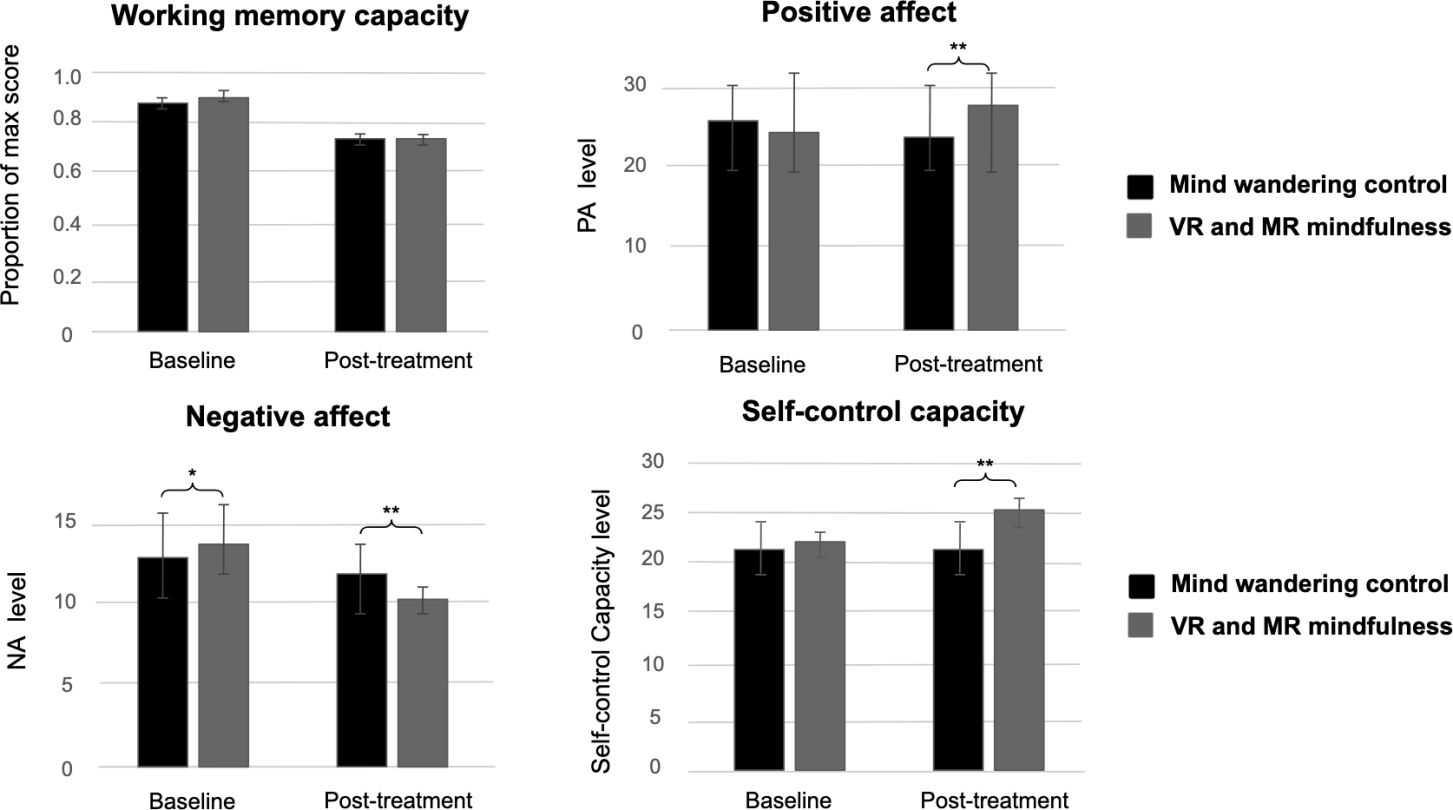
Working memory capacity (proportion of maximum score), positive affect, negative affect, and self-control capacity at baseline and post treatment across mind-wandering control and virtual reality and mixed reality mindfulness breathing conditions in a within-subjects experiment. Values indicate mean scores with error bars depicting SEs, which are consistent with the frequentist framework used for hypothesis testing. Total sample size was n=102. MR: mixed reality; NA: negative affect; VR: virtual reality.

Following our preregistered analytic plan, differences in working memory scores between conditions via a 2 (condition: VR+MR mindfulness vs mind-wandering)×2 (time: baseline vs posttreatment) were tested using repeated-measures ANOVA. A significant main effect of time on working memory was observed (*F*_1,101_=149.34, *η_p_*^2^=0.597; *P*<.001). However, there was no significant main effect of condition (*F*_1,101_=0.343, *η_p_*^2^=0.003; *P*=.56). Similarly, the interaction effect between condition and time on working memory capacity was not significant (*F*_1,101_=1.366, *η_p_*^2^=0.013; *P*=.25), and working memory scores differed similarly between baseline (mean 0.90, SD 0.13) and post treatment (mean 0.74, SD 0.18) in the VR and MR mindfulness breathing condition, and between baseline (mean 0.88, SD 0.15) and post treatment (mean 0.74, SD 0.17) in the mind-wandering control condition (Figure 7). This indicates that the difference in working memory performance between baseline and post treatment did not differ between the VR and MR mindfulness breathing condition and the mind-wandering control condition. Bayesian analysis further provided moderate evidence in support of the null hypothesis for both the main effect of condition and the time×condition interaction (BF_01_=7.42 and BF_01_=5.55, respectively), suggesting that VR and MR mindfulness breathing did not produce measurable improvements in working memory capacity compared to the control condition.

### PA

Consistent with our preregistered analytic plan, repeated-measures ANOVA was conducted to examine whether levels of PA were influenced by time and condition. First, no significant main effect of time on PA was observed after Bonferroni correction (*F*_1,101_=5.367, *η_p_*^2^=0.050; *P*=.02). This indicates that PA did not change significantly across time, regardless of the condition. However, a significant main effect of the condition on PA was found, *F*_1,101_=13.278, *η_p_*^2^=0.116; *P*<.001, indicating that the VR and MR mindfulness breathing condition showed significantly higher levels of PA (mean 26.47, SD 6.73) than the mind-wandering control condition (mean 24.20, SD 5.95). A Bonferroni correction was applied to account for multiple comparisons. With a corrected significance threshold of *P*<.0167 (ie, .05/3) for 3 comparisons, the main effect remained significant.

Most importantly, the interaction effect between condition and time on PA was significant, *F*_1,101_=58.30, *η_p_*^2^=0.366; *P*<.001, suggesting that the changes in PA scores over time differed significantly between the VR and MR mindfulness breathing condition and the mind-wandering control condition. Specifically, PA scores increased from baseline (mean 24.59, SD 6.74) to posttreatment (mean 28.35, SD 7.63) in the VR and MR mindfulness breathing condition, whereas scores in the mind-wandering control condition declined from baseline (mean 25.03, SD 6.24) to posttreatment (mean 23.36, SD 7.25), reflecting a statistically significant difference between conditions (Figure 7). This effect remained significant under the Bonferroni-corrected threshold (adjusted *P*<.0167). Bayesian analysis further provided extreme evidence supporting the alternative hypothesis (BF_10_>1000, BF_01_<0.001). The 95% credible intervals for the cell means indicated that PA decreased in the control condition from 25.03 (23.80-26.26) at baseline to 23.36 (21.94-24.79) post treatment, whereas it increased in the VR and MR mindfulness condition from 24.59 (23.26-25.91) to 28.35 (26.86-29.85).

### NA

A similar analytical approach was used to examine changes in NA. A significant main effect of time on NA was observed, *F*_1,101_=62.619, *η_p_*^2^=0.383; *P*<.001, suggesting that NA changed significantly from baseline to post treatment. While there was no main effect of the condition on NA, *F*_1,101_=1.384, *η_p_*^2^=0.014; *P*=.24, the interaction effect between condition and time on NA was significant (*F*_1,101_=39.087, *η_p_*^2^=0.279; *P*<.001). The results showed that the change in NA scores over time was significantly different between the VR and MR mindfulness breathing condition and the mind-wandering control condition. Specifically, NA scores declined from baseline (mean 14.15, SD 4.84) to post treatment (mean 10.58, SD 2.74) in the VR and MR mindfulness breathing condition, whereas the decline was less pronounced in the mind-wandering control condition from baseline (mean 13.15, SD 5.12) to post treatment (mean 12.36, SD 4.27). This effect remained statistically significant under the Bonferroni-corrected threshold (adjusted *P*<.0167). Bayesian analysis also provided extreme evidence in favor of the alternative hypothesis (BF_10_>1000, BF_01_<0.001). The 95% credible intervals indicated that NA decreased modestly in the control condition, from 13.15 (12.14-14.15) at baseline to 12.36 (11.52-13.20) post treatment, but showed a substantially larger decrease in the VR and MR mindfulness condition from 14.15 (13.20-15.10) to 10.58 (10.04-11.12).

### Self-Control Capacity

Similar analyses were conducted to examine changes in self-control capacity. A significant main effect of time was observed, *F*_1,101_=15.480, *η_p_*^2^=0.133; *P*<.001, indicating that self-control capacity changed significantly from baseline to post treatment. Moreover, a significant main effect of condition was found, *F*_1,101_=24.910, *η_p_*^2^=0.198; *P*<.001, and remained statistically significant after the Bonferroni correction. Consistently, the interaction effect between condition and time on self-control capacity was also statistically significant (*F*_1,101_=28.348, *η_p_*^2^=0.219; *P*<.001). The results showed that self-control capacity scores increased from baseline (mean 22.09, SD 4.60) to post treatment (mean 25.25, SD 4.42) in the VR and MR mindfulness breathing condition, whereas scores in the mind-wandering control condition decreased from baseline (mean 21.77, SD 5.14) to post treatment (mean 21.31, SD 5.46). Bayesian analysis further provided extreme evidence in favor of the alternative hypothesis (BF_10_>1000, BF_01_<0.001). Collectively, these findings suggest that exposure to VR and MR mindfulness breathing was associated with an improvement in self-control capacity.

## Discussion

Despite the growing body of research on mindfulness, much of the existing literature focuses on intensive, multisession interventions [46,78], leaving a gap in our understanding of the effectiveness of brief mindfulness practices. Some evidence suggests that even a single session of mindfulness can produce temporary benefits, but findings remain inconsistent, highlighting the need for more engaging and immersive approaches [42,60-62]. This study examined whether immersive VR technologies could enhance the effectiveness of brief mindfulness practices, potentially offering a more engaging and effective alternative. Brief mindfulness interventions often face challenges, such as limited attentional depth, insufficient repetition to consolidate self-regulatory skills, and inconsistent environmental contexts, that hinder transfer to daily life [66]. VR and MR-based delivery may help address these limitations by providing immersive environments that minimize distraction, provide sustained attentional focus, and simulate realistic emotional situations for practicing acceptance [66-68].

Consistent with our hypothesis, both VR and MR mindfulness breathing improved emotional well-being by increasing PA and reducing NA. These findings align with previous research showing that mindfulness breathing enhances PA [7,13,14] while alleviating depressive symptoms [7,101], stress [10,20,21], and anxiety [22,23,101]. According to the monitor and acceptance theory [56], mindfulness promotes emotion regulation through two core components: (1) attention monitoring, which heightens present-moment awareness; and (2) acceptance, which enables individuals to observe emotions without reacting impulsively. The immersive nature of VR and MR may further strengthen these processes by increasing engagement, reducing external distractions, and fostering a heightened sense of presence during mindfulness practice [66,102]. By creating a more absorbing and controlled environment, VR and MR may deepen both attention monitoring and acceptance, leading to greater emotional benefits [51,69]. It is also important to note that mindfulness aims to cultivate adaptive flexibility [34], allowing individuals to experience and regulate both positive and negative emotions in a context-sensitive manner. Our findings suggest that even a brief 15-minute mindfulness breathing session can enhance emotional well-being, adding to evidence that short-duration mindfulness interventions can yield immediate psychological benefits. Overall, these findings underscore the potential of VR and MR as effective tools for mood enhancement by strengthening core mindfulness processes. However, it is also possible that part of the emotional improvements observed may partly reflect the novelty and sensory engagement of immersive VR itself rather than mindfulness-specific mechanisms. Previous research shows that immersive VR can induce relaxation and PA through heightened presence and environmental realism, even without formal mindfulness instruction [67]. Thus, the affective benefits observed in this study may reflect a combination of novelty, attentional engagement, and mindfulness-related processes.

Beyond its benefits for emotional well-being, the study found that VR and MR mindfulness breathing also enhanced self-control capacity. Participants in the VR and MR conditions demonstrated greater self-control than those in the mind-wandering control condition, consistent with previous research showing that brief mindfulness practice can counteract self-control depletion [103,104]. This effect may stem from mindfulness enhancing 2 fundamental self-control processes: (1) emotion regulation [17-19] and (2) attention regulation [46,47,105], both of which are crucial for maintaining self-regulatory capacity [44,48]. The immersive qualities of VR and MR may further amplify these effects by deepening attentional absorption, minimizing external distractions, and alleviating cognitive strain. By reducing cognitive load, VR and MR mindfulness exercises may help preserve the mental resources necessary for self-control, thereby reinforcing self-regulatory functioning [44]. These findings contribute to the growing literature on mindfulness and self-control regulation and suggest that brief VR and MR mindfulness interventions could be useful for mitigating self-regulatory depletion [35,106]. Such interventions may be particularly suitable for individuals seeking to restore attentional and self-control resources during breaks in cognitively demanding environments, including workplaces and educational settings. By stabilizing attentional focus and alleviating cognitive strain, these interventions may enhance both productivity and well-being [52].

Contrary to our hypothesis, we found no significant difference in working memory between the VR and MR mindfulness and mind-wandering control conditions. This contrasts with previous research showing that mindfulness training can enhance working memory [32,33], suggesting potential limits to the immediate cognitive benefits of brief interventions in immersive VR and MR formats. These results highlight the need for caution when interpreting short-term cognitive outcomes following minimal exposure and are consistent with evidence that improvements in working memory typically emerge only after prolonged, repeated mindfulness training over multiple sessions [62,92]. Future research should therefore investigate whether extended VR and MR mindfulness programs yield cumulative cognitive gains as participants become more familiar and engaged with the practice.

Several factors may help to explain the limited cognitive benefits observed in our study. First, intervention duration and intensity are critical. Previous research indicates that mindfulness-based interventions typically require multisession practice to yield measurable cognitive benefits [33,42]. For example, studies in which participants engaged in 30‐45 minutes of mindfulness training over several weeks have reported measurable improvements in working memory [33,42]. In contrast, the single 15-minute session in our study may not have provided sufficient opportunity for participants to fully engage with the mindfulness practice or experience cognitive benefits. Moreover, working memory capacity is relatively stable and tends to change gradually rather than in response to brief interventions [107,108]. Given that working memory relies on complex neural processes that require continuous reinforcement [109,110], longer training durations may be necessary to induce measurable improvements. This account aligns with evidence that the cognitive benefits of mindfulness depend on repeated and prolonged engagement, which strengthens underlying attentional and working memory mechanisms [4,32,54,55]. Future research should therefore explore whether extended VR and MR mindfulness interventions produce cumulative cognitive gains over time. It will be important to determine whether repeated practice enhances working memory or whether habituation results in reduced engagement. Clarifying these long-term dynamics is crucial for evaluating the feasibility of VR and MR-based mindfulness as a long-term cognitive intervention.

An alternative explanation for the lower working memory scores observed post treatment relative to baseline is the influence of uncontrolled stressors or fatigue during the study period. Academic workload, sleep disruption, or daily emotional stress may have temporarily impaired prefrontal functioning and reduced attentional capacity during testing [111-113]. Previous research indicates that acute stress can disrupt working memory by diverting cognitive resources from executive processes [114]. Repeated testing may also have contributed to cognitive fatigue, particularly given the demanding nature of the complex span tasks [109]. The comparable pattern observed across both conditions suggests that these differences reflect general influences rather than effects of the experimental manipulation itself.

Beyond intervention duration and intensity, the environmental context of mindfulness practice may also influence cognitive outcomes. While previous studies have often used nature-based settings (eg, forests, rivers, and beaches) for mindful practice, which have been known to promote relaxation and reduce cognitive load [66,115], our study used VR metaverse and MR environments. Although VR and MR provide immersive experiences, they may also introduce additional cognitive demands, such as the need to navigate virtual interfaces, which could detract from mindfulness engagement, particularly for individuals unfamiliar with the technology [82]. This may help explain why VR and MR did not yield measurable cognitive improvements; compared with natural environments, immersive virtual settings may not provide the restorative conditions necessary for cognitive recovery [92], potentially contributing to the null findings in our study.

While this study highlights the potential psychological benefits of VR and MR mindfulness interventions, several limitations should be considered. First, future research should include more diverse samples to enhance generalizability beyond young adults. Younger participants may differ from older adults or clinical populations in their adaptability to emerging technology and stress profiles [78]. Including individuals from a wider range of age groups, occupational backgrounds, and mental health conditions would provide a more comprehensive understanding of the effectiveness of VR and MR mindfulness interventions.

Second, the working memory assessment in this study may be subject to task impurity [116,117]. We used the OSpan task at baseline and the RotSpan task post treatment, which engage different cognitive processes (eg, verbal vs spatial processing) [83]. Relying on only 2 complex-span tasks may have provided an incomplete evaluation of working memory capacity, making the null findings potentially task-specific rather than reflective of actual cognitive changes. In addition, we did not include tasks from other paradigms, such as N-back or updating tasks, which assess complementary components of working memory (eg, tracking, updating, and replacing information) [108]. Future research should incorporate a broader set of complex span tasks to improve construct validity.

Third, the study did not account for individual differences in stress exposure, fatigue, or sleep quality across sessions. Variability in these uncontrolled factors may have contributed to fluctuations in working memory performance, underscoring the need for future studies to monitor or standardize participants’ stress levels across testing periods [118]. Another limitation concerns participants’ familiarity with VR technology. Because our sample primarily consisted of young adults, their higher levels of technological comfort may have facilitated immersion and engagement during the intervention. These effects may not generalize to older adults or clinical populations with limited VR experience. Future research should therefore assess VR familiarity as a potential moderator and examine whether individual differences in technological comfort influence intervention outcomes.

Finally, the absence of a VR-based control condition (ie, a nonmindfulness VR control condition) presents another limitation. We selected a mind-wandering task as the control condition to ensure that participants engaged in a cognitively active but nonmindful activity, thereby providing a clearer contrast with the mindfulness intervention [43]. Although this design differentiates mindfulness-specific effects from general cognitive engagement, it does not fully disentangle the potential contribution of VR immersion itself from those of mindfulness practice. Future research should include an appropriate VR control condition to clarify the distinct contributions of immersion and mindfulness.

In summary, this study examined the effects of VR and MR mindfulness on cognitive and emotional outcomes relative to a mind-wandering control condition. While no cognitive improvements were observed, the findings underscore the potential of VR and MR mindfulness in enhancing emotional well-being, consistent with research suggesting that immersive environments may facilitate psychological benefits [71,79]. In comparison to existing studies that have primarily focused on stress reduction or relied on multisession digital interventions [46,78], this study demonstrates that a single brief VR or MR session can enhance key emotional and self-regulatory outcomes. As such, these findings underscore the potential of VR and MR mindfulness applications as innovative and scalable tools for promoting mental well-being. As these technologies become increasingly incorporated into mental health interventions, future research should investigate their long-term efficacy and applicability across diverse populations and identify the specific features, such as sensory immersion, attentional focus, and guided engagement, that optimize their therapeutic potential. Furthermore, the declining cost and increasing accessibility of stand-alone VR headsets (eg, Meta Quest) increase the feasibility of implementing immersive mindfulness programs in organizational and educational settings [68]. Despite initial setup and training demands, these interventions can be cost-effective over time because the same digital content can be reused across participants with minimal supervision. Their scalability and portability make them suitable for brief restorative sessions that enhance attention and emotional well-being [92]. Future research should therefore examine implementation feasibility, including usability, cost-benefit considerations, and long-term adherence, to determine the broader applicability of VR- and MR-based mindfulness interventions. By leveraging the unique affordances of immersive technology, VR and MR mindfulness may provide innovative and accessible approaches to enhancing emotional regulation and well-being across clinical, educational, and workplace settings.

## Abbreviations

JARS: Journal Article Reporting Standards
MBSR: mindfulness-based stress reduction
MR: mixed reality
NA: negative affect
OSpan: operation span
PA: positive affect
RotSpan: rotation span
VR: virtual reality
